# Transperitoneal Ligature of the Internal Iliac Artery

**Published:** 1902-08-23

**Authors:** 


### TRANSPERITONEAL LIGATURE OF THE INTERNAL ILIAC ARTERY.

Mr. Herbert Page[1] records a case of rupture of gluteal aneurism in which it became necessary to tie the iliac artery, this being done by the transperitoneal method. He says that the rupture of a gluteal aneurism can never fail to be of interest to surgeons, because of the classical case in which the great Syme pointed out for all time the way to deal with it. Circumstances may, however, at any moment render it impossible, in the course of an operation for turning out the clot of such an aneurism, to be content with applying a ligature to the bleeding gluteal artery, even though the bleeding be stayed thereby, and the surgeon may find it imperative, as in the case related, to tie the feeding trunk. The patient, a man aged 44 but looking much older, had been ill five weeks with a swelling of the right buttock, which, on admission to St. Mary's Hospital, was of huge size reaching from the crest of the ilium to below the great trochanter. There was some inflammatory redness, and the house surgeon, Mr. A. Whitmore, made a small inch-long incision, but finding only blood clot closed the wound. At the operation which was subsequently performed a large incision was made, and upwards of two pints of clot were removed. The conditions which led to the necessity for tying the iliac artery are thus described by Mr, Page : "Deeper clots and laminated fibrin being detached it was now for the first time obvious that active hæmorrhage was taking place from the sciatic notch. This was controlled by sponge pressure until all clot had been quickly removed, and we were then able to see that the blood came in spurts from within the sciatic notch, evidently from the gluteal artery itself. Happily, this bleeding was under complete control by the finger, but the actual bleeding point could not be seen, and evidently lay deeper on the inner side of the bone. After several unsuccessful attempts to seize the bleeding point with pressure forceps, Mr. Silcock ultimately made a lucky hit with a pair of forceps bent at the end to a right angle and completely stopped the bleeding." It seemed, however, that no reliance could be placed upon these forceps for the permanent arrest of hæmorrhage, since they might easily be displaced by any accidental movements. With the patient still lying on his side a ligature was applied to the common iliac artery by the transperitoneal method. So far as hæmorrhage was concerned the treatment was effectual. Unfortunately the patient died of pneumonia, possibly of septic origin, five days after the operation.

[1] Lancet, Aug. 16.

